# Aminoguanidine inhibits aortic hydrogen peroxide production, VSMC NOX activity and hypercontractility in diabetic mice

**DOI:** 10.1186/1475-2840-8-65

**Published:** 2009-12-30

**Authors:** Jeong-Ho Oak, Ji-Youn Youn, Hua Cai

**Affiliations:** 1 Department of Anesthesiology, David Geffen School of Medicine at University of California Los Angeles (UCLA), Los Angeles, CA, USA; 2 Department Medicine, David Geffen School of Medicine at University of California Los Angeles (UCLA), Los Angeles, CA, USA

## Abstract

**Background:**

Dysfunctionally uncoupled endothelial nitric oxide synthase (eNOS) is involved in producing reactive oxygen species (ROS) in the diabetic endothelium. The present study investigated whether anti-diabetes drug Aminoguanidine (AG) has any effect on eNOS function and vascular oxidant stress.

**Methods and Results:**

Blood glucose levels were increased to 452.0 ± 15.1 mg/dl in STZ-treated male C57BL/6J mice (148.4 ± 3.2 mg/dl in untreated controls). Aortic productions of NO^• ^and O_2_^•- ^were measured specifically and sensitively using electron spin resonance. Diabetic mice had a marked increase in aortic O_2_^•- ^production. Aortic hydrogen peroxide (H_2_O_2_) production was also increased in diabetic aortas and significantly attenuated by AG. AG however had only a marginal effect in reducing aortic O_2_^•- ^production, which corresponded to a minimal effect in improving aortic nitric oxide (NO^•^) bioavailability. The endothelium-dependent vasodilatation however was modestly but significantly improved by AG, likely consequent to AG-induced reduction in hyper-contractility. NAD(P)H oxidase (NOX)-dependent O_2_^•- ^production was completely attenuated by AG in endothelium-denuded diabetic aortas.

**Conclusion:**

In summary, despite that AG is not an effective eNOS recoupling agent presumably consequent to its ineffectiveness in preventing endothelial NOX activation, it is inhibitory of aortic H_2_O_2 _production, VSMC NOX activity, and hypercontractility in diabetes.

## Background

Cardiovascular complications are the primary causes of mortality in diabetic patients [1,2]. Accumulating evidence has demonstrated that increased production of reactive oxygen species (ROS) contributes to etiology of diabetes [3-6] and its cardiovascular complications [4,6-11]. Various enzymatic systems have been shown responsible for diabetic oxidant stress, including xanthine oxidase [12], NAD(P)H oxidase [13,14], and the more recently established, uncoupled endothelial nitric oxide synthase (eNOS) [15,16]. Oxidant stress contributes to diabetic vascular damages by acceleration of advanced glycation end products (AGEs) formation, modulation of extracellular matrix proteins, promotion of cell proliferation and migration, stimulation of kinases and proinflammatory proteins, and importantly, inactivation of nitric oxide (NO^•^), all of which are closely associated with the pathogenesis of diabetic vascular complications [17,18].

Aminoguanidine (AG) is one of the most extensively used inhibitors of AGEs accumulation. Beneficial effects in preventing cardiovascular events in diabetic rats have been observed with AG treatment, likely attributed to its effects on stopping AGE formation [19]. Besides its inhibitory action on AGE formation, AG acts as a competitive and selective inhibitor for inducible nitric oxide synthase (iNOS) [20]. This action of AG has been known to be associated with reduction of peroxinitrite (ONOO-), which has deleterious roles in inducing NO^• ^deficiency and cellular damages through degradation of eNOS cofactor, and inductions of inflammation, lipid peroxidation, protein nitrosylation and DNA fragmentation [18,21,22]. Previous investigations have also demonstrated that AG reduced hydrogen peroxide (H_2_O_2_) induced intracellular hydroxyl radical formation and apoptosis, further demonstrating a potential antioxidant activity [23,24]. These multiple actions of AG may improve endothelial function in diabetes independent of its AGE-inhibiting activity [22,23]. Apart from its beneficial effects, high dose of AG is associated with some adverse effects such as autoimmune symptoms, abnormal liver function, gastrointestinal disturbance, and flu-like symptoms [25,26]. These side effects are likely related to its structural similarities to hydrazine, an inducing factor of lupus like syndrome, and L-arginine, a substrate of NO^• ^synthase [27]. Thus the potential effect of AG is complex in diabetes associated cardiovascular complication. The direct impact of AG on aortic oxidant stress and eNOS function is completely unknown despite that AG was found to suppress superoxide (O_2_^•-^) production, mitochondrial complex III activity and eNOS uncoupling in the kidney [26,28].

Therefore, in the present study we treated STZ-induced diabetic mice *in vivo *with AG, and measured aortic O_2_^•- ^and NO^• ^productions by electron spin resonance (ESR) sensitively and specifically. AG only marginally reduced total aortic O_2_^•- ^production although it significantly attenuated aortic hydrogen peroxide (H_2_O_2_) generation. Endothelium-dependent vasodilatation was modestly yet significantly improved which was accompanied by AG-dependent significant reduction in aortic hypercontractility. NAD(P)H oxidase (NOX)-dependent O_2_^•- ^production in endothelium-denuded aortas was significantly attenuated by AG, likely contributing to the reduction in phenylephrine (PE)-induced hypercontractility. These data seem to implicate that although AG is ineffective in recoupling eNOS in diabetic aortas, it reduces vascular H_2_O_2 _production and hypercontractility in diabetes, which may in part account for its beneficial effects in preventing vascular disease development.

## Methods

### Diabetic mice and drug interventions

Male C57BL/6J mice (6-8 weeks old) were obtained from Jackson Laboratories. Mice were housed in a pathogen-free condition. The Institutional Animal Care and Usage Committee at the University of Chicago and University of California Los Angeles approved the use of animals and experimental procedures. Diabetes was induced by tail vein injection of Streptozocin (STZ, 100 mg/Kg) dissolved in 50 μL of 0.9% saline immediately before use, once a day for three days [15,29,30]. Blood glucose was determined using the One Touch Ultra^® ^blood glucose meter (Lifescan) at baseline and on day four post STZ injection for each individual mouse. On day four, STZ diabetic mice were injected with Aminoguanidine (AG) [31] dissolved in 0.9% saline via tail vein at 100 mg/kg/day for three days. By day seven, animals were sacrificed using CO_2 _inhalation and whole aorta was removed, cleared from surrounding connective tissues and cut transversely into 2 mm or 3 mm rings for subsequent experiments. Nitric oxide production and vasoreactivity measurement was performed with 2 mm of aorta segments and determination of superoxide and hydrogen peroxide level was conducted with each 3 mm of aorta. This model of diabetes is characterized by acute hyperglycemia. No renal dysfunction occurs during the study period of seven days [32].

### Electron spin resonance measurement of aortic superoxide production

Freshly isolated aortas were placed into chilled modified Krebs/HEPES buffer (composition in mmol/L: 99.01 NaCl, 4.69 KCl, 2.50 CaCl_2_, 1.20 MgSO_4_, 1.03 KH_2_PO_4_, 25.0 NaHCO_3_, 20.0 Na-HEPES, and 5.6 glucose [pH 7.4]), cleaned of excessive adventitial tissue, with care taken not to injure the endothelium. The specific O_2_^•- ^spin trap methoxycarbonyl-2,2,5,5-tetramethyl-pyrrolidine (CMH, 500 μmol/L, Alexis) solution was prepared freshly in nitrogen gas bubbled Krebs/HEPEs buffer containing diethyldithiocarbamic acid (DETC, 5 μmol/L Sigma) and deferoxamine (25 μmol/L, Sigma). Aortic segment (~3 mm) was then mixed with the spin trap solution and loaded into glass capillary (Fisher Scientific) for analysis of O_2_^•- ^signal (CM^• ^formed after trapping O_2_^•-^) using the electron spin resonance (ESR) spectrometer (Miniscope MS200, Magnettech, Germany). Some of the intact or endothelium-denuded aortic segments were incubated in presence or absence of NSC23766 (200 nmol/L, 90 min) to detect NOX sensitive superoxide production. The ESR settings used were bio-field, 3350; field sweep, 45.00 G (1 G = 0.1 mT); microwave frequency, 9.78 GHz; microwave power 7 dB (20 mW); modulation amplitude, 3000 mG; 4,096 points of resolution; and receiver gain, 700.

### Amplex-Red assay for hydrogen peroxide production

Freshly isolated aortic rings (4 × 2 mm) were used for assessment of H_2_O_2 _production using a fluorometric horseradish peroxidase assay (Amplex-Red assay, Molecular Probes). Fluorescence was measured (excitation 530 nm and emission 590 nm) after 1 hour incubation at 37°C in dark against background fluorescence of buffer. Polyethylene glycol conjugated catalase (PEG-CAT, 300 U/ml, Sigma)-inhibitable fraction reflects specific H_2_O_2 _signal. The rate of H_2_O_2 _production was presented as pmol/mg protein/min after calculation according to a standard curve generated using fresh H_2_O_2 _in reaction buffer [33].

### Electron spin resonance of aortic nitric oxide production

Freshly isolated aortic rings (6 × 2 mm) were incubated with freshly prepared NO^•^-specific spin trap Fe^2+^(DETC)_2 _(0.5 mmol/L) in modified Kreb's HEPES buffer (KHB) at 37°C for 60 min [spin trap and buffer recipe see above and previous publication [34], in the presence or absence of calcium ionophore A23187 (10 μmol/L). After the incubation, the aorta in KHB was snap-frozen in liquid nitrogen and loaded into a finger Dewar for analysis with ESR spectrophotometer. The instrument settings were as the followings: bio-field, 3280; field sweep, 77.54 G (1 G = 0.1 mT); microwave frequency, 9.78 GHz; microwave power, 4 dB (40 mW); modulation amplitude, 10 G; 4,096 points of resolution; and receiver gain, 900.

### Assessment of vascular reactivity

Freshly prepared aortic rings (2 mm) were placed in organ baths containing modified Kreb's HEPES buffer(recipe see above), aerated with a mixture of 95% oxygen/5% carbon dioxide and maintained at 37°C. After being kept under 5 mN tension for 90 min to stabilize, cumulative tension was measured by a Graz Tissue Bath System (Hugo Sachs Elektronik/Harvard Apparatus GmbH, March Hugstetten, Germany) connected to a The MP100 workstations (BioPac Systems). Relaxation curve to acetylcholine (10^-9 ^to 10^-6 ^M) were assessed in aortic segment after contraction by phenylephrine (PE, 5 μmol/L). Data acquisition process and post-acquisition calculations were performed with AcqKnowledge software (BioPac Systems).

### Statistical analysis

Differences among different groups of means were compared with unpaired *t*-test for two means and ANOVA for multiple means. Statistical significance was set for p < 0.05. All grouped data shown in the figures were presented as mean ± SEM.

## Results

### Effect of Aminoguanidine on hyperglycemia

Diabetic mice were created by streptozotocin (STZ) administration. On day of harvest (7-8^th ^day after initial STZ injection, same hereafter), blood glucose was elevated to 452.0 ± 15.1 mg/dl in diabetic mice vs 148.4 ± 3.2 mg/dl in the C57BL6 controls. AG (100 mg/kg/day, same hereafter) treatment since day 4 had no significant effect on STZ induction of hyperglycemia (data not shown).

### Effect of Aminoguanidine on aortic superoxide production

**Figure 1:**
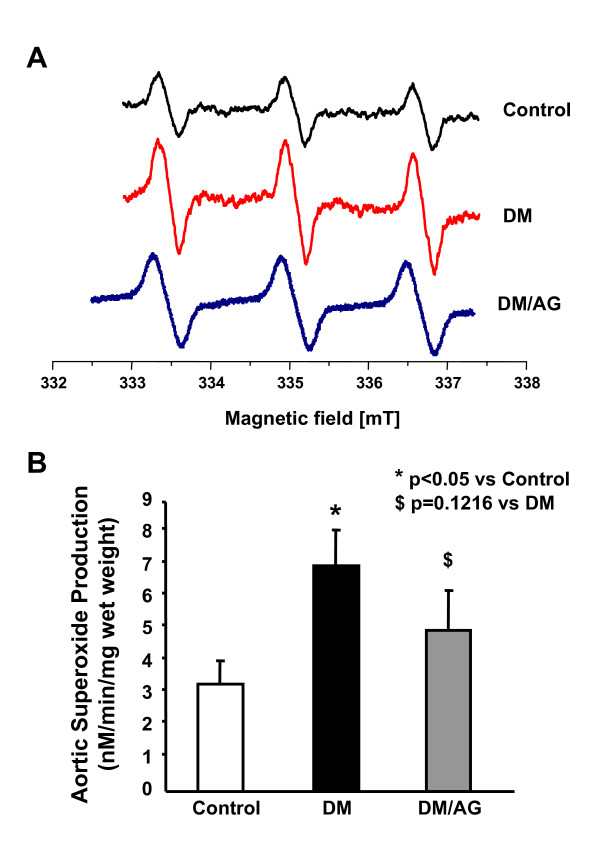
**Effects of AG on aortic superoxide (O_2_^•-^) production**. A: Representative spectra for aortic O_2_^•- ^detected by ESR. Freshly isolated aortic segments (~3 mm) were incubated with spin trapping solution and then analyzed using ESR. B: Grouped data of aortic O_2_^•- ^production expressed as nmol/L per min per mg wet weight. Data are presented as mean ± SEM, n = 6-8.

Aortic production of O_2_^•-^, etected specifically by electron spin resonance (ESR) and a cell-permeable specific spin trap, was more than doubled in diabetic mice (control vs diabetics: 3.3 ± 1.6 vs 7.0 ± 2.6 nmol/L per min per mg wet weight of aorta, p < 0.05). AG attenuated this response however marginally and insignificantly, as demonstrated by both representative ESR spectra and grouped data (Figs. 1A&B).

### Effect of Aminoguanidine on aortic hydrogen peroxide production

**Figure 2:**
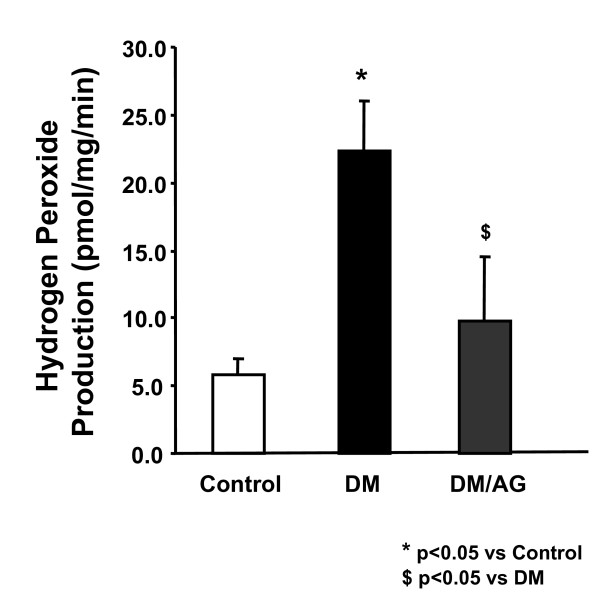
**Effects of AG on aortic hydrogen peroxide (H_2_O_2_) production**. Total aortic H_2_O_2 _production by Amplex Red Assay. Data are presented as mean ± SEM, n = 8.

Aortic H_2_O_2 _was detected specifically using an Amplex Red Assay (details see Methods section). Diabetic mice had a more than 4-fold increase in H_2_O_2 _production (5.86 ± 1.21 vs 22.39 ± 3.61 pmol/mg protein/min for control vs diabetics), which was significantly attenuated by treatment with AG (9.77 ± 4.71 pmol/mg protein/min, Fig. 2A, p < 0.05).

### Aminoguanidine failed to restore aortic NO^• ^production

**Figure 3:**
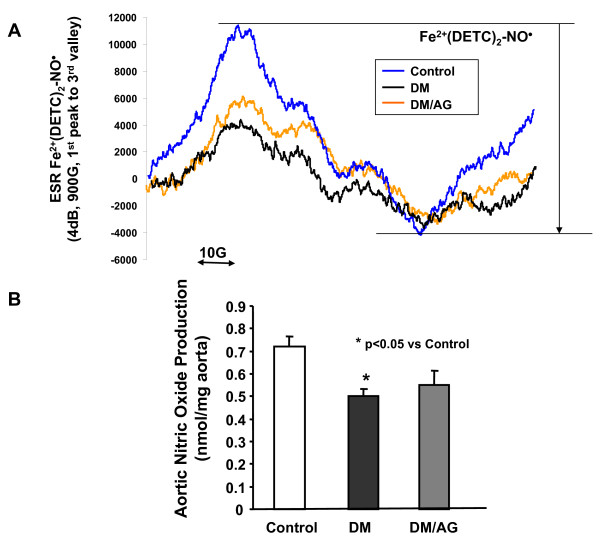
**Effects of AG on aortic nitric oxide (NO^•^) bioavailability in diabetes**. **A**: Representative ESR spectra for NO^•^; **B**: Grouped data of bioavailable NO^•^. Data are presented as mean ± SEM, n = 6.

Aortic NO^• ^production was directly and characteristically detected using ESR. As shown in representative ESR spectra and grouped data (Figs. 3A&B), diabetic mice had markedly reduced bioavailable NO^• ^(0.50 ± 0.08 in diabetes vs 0.72 ± 0.10 nmol/mg dry weight) and this response was however not significantly affected by treatment with AG (0.55 ± 0.15 nmol/mg dry weight). This result indicated that AG was ineffective in fully restoring eNOS function.

### Aminoguanidine partially restored endothelium-dependent vasorelaxation: Role of attenuation of hypercontractility?

**Figure 4:**
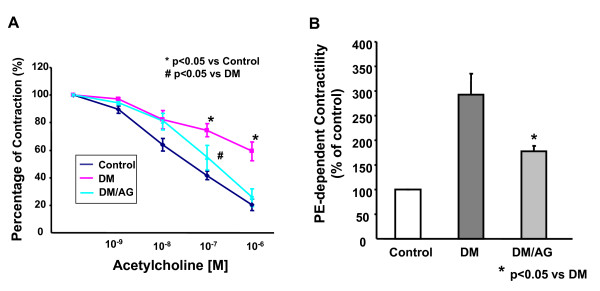
**Effects of AG on vascular reactivity**. **A**: AG partially restored endothelium-dependent vasorelaxation. **B**: AG diminished diabetes induced aortic hypercontractility. Data are presented as mean ± SEM, n = 6.

**Figure 5:**
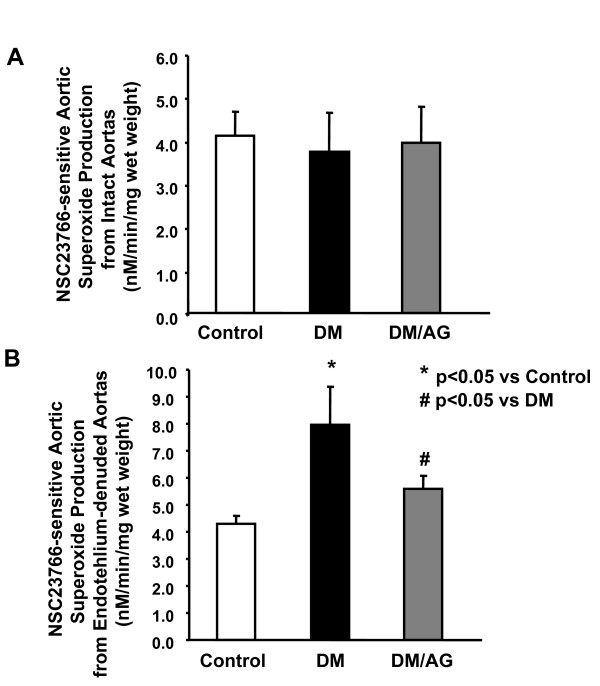
**Effects of AG on NOX-sensitive superoxide (O_2_^•-^) production**. **A**: NSC23766-sensitive O_2_^•- ^production from intact aortas **B**: NSC23766-sensitive O_2_^•- ^production from endothelium denuded aortas. Data are presented as mean ± SEM, n = 6.

Interestingly, although AG did not protect NO^• ^bioavailability likely due to its insignificance in reducing O_2_^•- ^production from eNOS, AG partially yet significantly restored endothelium-dependent vasorelaxation (Fig. 4A). Intriguingly, diabetic aortas exerted a more than 3-fold increase in basal contractility in response to PE, which was markedly attenuated by AG (Fig. 4B). Vascular smooth muscle cell (VSMC) production of O_2_^•- ^has been implicated in the hypercontractile response in diabetic blood vessels [35,36]. Furthermore, the source of this O_2_^•- ^production could be NAD(P)H oxidase (NOX) [35]. Previously we have successfully measured O_2_^•- ^production from endothelium-denuded vessels in the presence of NOX inhibitor. As shown in Fig. 5, consistent to previous findings, NOX remained active in VSMC [16]. AG completely diminished NSC23766-sensitive O_2_^•- ^production, indicating that attenuation of NOX may have accounted for reduced hypercontracility observed with AG, which was further linked to improved vasorelaxation.

It is important to emphasize that in the intact endothelium, uncoupled eNOS is the primary source of ROS production 7 days after STZ injection [16]. This is presumably consequent to a transient activation of endothelial NOX based on our *in vitro *data from cultured aortic endothelial cells [34]. In this earlier study we found that, via transient activation endothelial NOX, angiotensin II (Ang II) induces H_2_O_2 _dependent eNOS uncoupling. Indeed, we found that *in vivo *treatment with Ang II signaling attenuators candesartan or captopril completely prevented eNOS uncoupling in diabetes [16].

We also found that after removal of endothelium to allow sufficient spin trap penetration to the underneath VSMC, NOX-dependent O_2_^•- ^production remained elevated by day 7, which was found attenuated by candesartan or captopril previously, and now by AG. Therefore we believe that although AG may not have any effects on the endothelial NOX isoform in contrast to the Ang II attenuators, it is effective in inhibiting the VSMC NOX isoform. We are working on follow-up studies to identify cell-specific NOX isoforms that are involved in the eNOS uncoupling and NOX activation in diabetic endothelial cells and VSMC. These would be however beyond the scope of the present study.

## Discussion

The present study systematically studied effects of AG on vascular oxidant stress, eNOS function and endothelium-dependent vasorelaxation. Whereas AG only partially reduced vascular O_2_^•- ^production, it attenuated H_2_O_2 _production significantly and improved endothelium-dependent vasodilatation, likely via a reduction in NOX-linked hypercontractility. Although AG does not seem to be an effective eNOS recoupling agent like Ang II signaling attenuators [16], it may still exert beneficial effects via attenuating VSMC NOX activity and H_2_O_2 _production.

Oxidant stress has been implicated in micro and macrovascular complication of diabetes [37]. Moreover, excessive generation of O_2_^•- ^in endothelium by hyperglycemia has been considered one of the major factors involved in accelerating vascular complications. Recent studies have shown that eNOS uncoupling is the primary source of O_2_^•- ^production in the diabetic endothelium [15,16], whereas NAD(P)H oxidase remain active in sub-endothelial VSMC [16]. eNOS uncoupling is a phenomenon whereby the enzyme generates O_2_^•- ^rather than NO^•^. Previously we established that increased O_2_^•- ^production in STZ-induced diabetic mice is attributed to eNOS uncoupling, which was significantly attenuated by Ang II signaling blockers [16]. In the present study we examined effects of AGE chain breaker AG in recoupling eNOS and found AG failed to significantly reduce aortic O_2_^•- ^production in diabetes. AG also failed to significantly restore aortic NO^• ^bioavailability. AG has been used as a iNOS inhibitor because of its structure similarity with L-arginine; and it is also known as a weaker inhibitor for eNOS [27,38]. Whether or not these are linked to the ineffectiveness of AG in restoring NO^• ^production however, remain unclear.

Despite lack of significant impacts on O_2_^•-^/NO^• ^pathway, AG significantly attenuated aortic H_2_O_2 _production in diabetic mice. It also significantly, though modestly, improved endothelium-dependent vasodilatation, which is likely consequent to a reduction in hypercontractility that is associated with attenuation of VSMC NOX activity. Ineffective eNOS recoupling agent that is, AG proved to be highly effective in attenuating diabetes-induced aortic hypercontractility.

There have been controversial observations of diabetic hypercontracility given species, age, diabetic type, diabetic stage, and vessel types [14,39-43]. For instance, a recent study by Su *et al*., showed that acetylcholine dependent dilation was decreased in diabetes whereas there was no difference between control and diabetic group in response to PE, concluding no specific role of VSMC contraction in type 2 diabetes [14]. Not in agreement with our data, they observed that AG did not alter vasocontraction to PE, indicating that AGE formation is not associated with muscle contraction in type 2 diabetic mesenteric arteries [14]. On the other hand, other studies demonstrated hypercontractility to PE in aortas of STZ-induced diabetes [44,45]. In agreement with our results, AG normalized the response to PE in STZ diabetic mice without affecting plasma glucose levels [44]. Taken together, our results are consistent with previous observations regarding effect of AG on vascular hypercontraction in conduit vessels of early stage type 1 diabetes, hence indicating a potential beneficial effect of AG for prevention of cardiovascular complication for this particular diabetic type and stage.

It has been hypothesized that the basal hypercontractility is dependent on O_2_^•- ^in STZ induced diabetic mice [36]. In the denuded aortas of human diabetes, O_2_^•- ^production was also found elevated [35]. Indeed we have observed that in the endothelium-denuded aortas, AG completely attenuated NOX dependent O_2_^•- ^production from VSMC (Fig. 5). Theoretically this reduction in O_2_^•- ^could feed back to the endothelium to partially contribute to preservation of NO^• ^bioavailability. On the other hand, loss of O_2_^•- ^in VSMC may directly modulate VSMC contractility via undefined mechanisms. It is interesting to speculate that loss of NOX-derived O_2_^•- ^in diabetic VSMC might underlie AG reduction of hypercontractility.

## Conclusion

In summary, AG has been examined systematically for its effects on vascular oxidant stress, eNOS function and endothelium-dependent vasorelaxation. To our knowledge these are first endeavors. This agent was ineffective in reducing plasma glucose levels, partially effective in inhibiting total O_2_^•-^, and insignificantly effective in improving NO^• ^bioavailability in diabetes. However, it reduced aortic H_2_O_2 _production and improved endothelium-dependent vasorelaxation while diminished hypercontractility of aortas. Whether this is attributed to AG-dependent significant reduction in VSMC NOX activity remains to be further elucidated.

## Competing interests

The authors declare that they have no competing interests.

## Authors' contributions

JHO collected data and performed data analysis. JYY participated in data analysis and manuscript preparation. HC designed the study and participated in data analysis and interpretation, and manuscript preparation. All authors have read and approved the final version of the manuscript.
